# Differences in Clinicopathological Features, P16Ink4a and P57KIP2 Immunohistochemical Expressions, and Survival Between Colorectal Carcinoma in Rectosigmoid and Other Colonic Locations

**DOI:** 10.7759/cureus.62061

**Published:** 2024-06-10

**Authors:** Fatma Alzahraa A Elkhamisy, Elshaimaa A Aboelkomsan, Abd AlRahman M Foda

**Affiliations:** 1 Pathology Department, Faculty of Medicine, Helwan University, Cairo, EGY; 2 Pathology Department, School of Medicine, Newgiza University, Giza, EGY; 3 Pathology Department, Faculty of Medicine, Mansoura University, Mansoura, EGY; 4 Pathology Department, General Medicine Practice Program, Batterjee Medical College, Jeddah, SAU

**Keywords:** target proteins, cyclin-dependent kinase inhibitors, personalized therapy, gastrointestinal pathology, survival, rectosigmoid, colorectal cancer, p57, p16

## Abstract

Background

One unique criterion of colorectal carcinoma (CRC) is the different locations within the colorectum. Different CRC sidedness/locations could have distinct criteria, including risk factors, morphological features, genetic alterations, prognostic factors, and clinical outcomes. Nearly half of the CRC cases occur in the rectal-sigmoid locations, while other colonic locations constitute the other half. Investigating specific protein expression patterns in the rectosigmoid CRC (rsCRC) compared to other colonic (ocCRC) locations helps understand the disease pathogenesis, predict prognosis, and design personalized treatments. This study is the first to compare P16Ink4a and P57KIP2 immunohistochemical (IHC) expression in rsCRC to ocCRC and examine their relationship to disease outcomes in both locations.

Materials and methods

A comparative cross-sectional study used tissue microarray slides from rsCRC and ocCRC that were immunohistochemically stained by anti-P16Ink4a and P57KIP2 antibodies. A semi-quantitative scoring system classified each marker's expression as positive or negative. The statistical analysis compared clinicopathological features, P16Ink4a and P57KIP2 expressions, and their relationship to clinical outcomes in rsCRC and ocCRC cases.

Results

One hundred fifty CRCs were distributed into the rsCRC cases (n=86, 57.3%) and the ocCRC cases (n=64, 42.7%). The rsCRC cases had a significantly lower age <40 years (P=0.002), higher frequency of mismatch repair (MMR) proficient status (P=0.003), and perineural invasion (P=0.008), with lower disease-free (DFS) and overall survival (OS) (P=0.03, and P=0.015, respectively). Significantly higher positive P16Ink4a and P57KIP2 IHC expressions were found in the rsCRCs compared to the ocCRCs (P=0.02, and P=0.03, respectively); however, their relationship to the hazards (HR) of recurrence (HR=4.02, P=0.058, and HR=0.36, P=0.14, respectively) and mortality (HR=2.56, P=0.21, and HR=0.23, P=0.58, respectively) in the rsCRC group was statistically nonsignificant. In the ocCRC group, P16Ink4a positivity was significantly associated with a higher disease recurrence and mortality hazard (HR=8.19, P=0.007, and HR=5.57, P=0.037, respectively), while P57KIP2 positivity was significantly associated with a lower mortality hazard (HR=0.12, P=0.027).

Conclusion

The rsCRCs differ from ocCRCs in clinicopathological criteria and protein expression patterns. Though P16Ink4a and P57KIP2 IHC expressions are higher in the rsCRC than in the ocCRC, their value as outcome predictors is higher in the ocCRCs rather than the rsCRCs. P16Ink4a and P57KIP2 can act as prognostic markers and be suitable targets for therapy modulation in the ocCRC group.

## Introduction

Colorectal carcinoma (CRC) is one of the leading causes of human cancer deaths worldwide [1]. It is a heterogeneous group of neoplasms rather than a neoplasm with unified criteria; hence, extensive investigation of the unique features of different CRCs is needed to understand the disease better and plan personalized treatments [2]. One unique criterion of CRCs is the different cancer locations within the colon [3]. Literature showed that different CRC sidedness/location within the colorectum could have distinct criteria, including risk factors, morphological features, genetic alterations, prognostic factors, and clinical outcomes [3,4].

The rectal-sigmoid (i.e., rectosigmoid) CRC incidence exceeds 50% of CRC cases in some epidemiological studies [5,6]. The rectosigmoid location for cancers has recently gained more focus for investigation due to its therapeutic and prognostic challenges [7,8]. Investigating specific protein expression patterns in the rectosigmoid CRC (rsCRC) compared to other colonic (ocCRC) locations helps understand the pathogenesis of these tumors, predict prognosis, and design personalized treatments according to CRC location.

Two families of cell cycle regulators that suppress cell proliferation through inhibiting cyclin-dependent kinases (CDKs) have been identified [9,10]. First, the INK4 family inhibits CDK4, with P16Ink4a as a member. Second, the CDK interacting protein/kinase inhibitory protein (CIP/KIP) family includes P57KIP2 as a member [9,10]. Alterations in P16Ink4a and P57KIP2 proteins (referred to as P16 and P57, respectively, in the current study) have been described in different cancers [11-13]. Loss of either protein expression through deactivation has been linked to CRC carcinogenesis [14,15]. Moreover, P16 expression has been linked to human papillomavirus (HPV) infection in CRC [16]. P16's precise association with clinicopathological data and survival probability in CRC is still questionable [15,17].

Recently, targeted modulation of the P16 and P57 expression in cancers has been investigated with promising initial results [10,18]. Differential expressions of P16 and P57 in the subsets of the same cancer have been recently studied and have yielded specific patterns in some cancer types [10,13].

The differential P16 and P57 expression in rsCRC compared to other CRC colonic locations has not been investigated yet. This study aims to compare P16 and P57 immunohistochemical (IHC) expression in rsCRC to ocCRC and examine their relationship to disease outcomes in both locations. Additionally, it compares the clinicopathological characteristics of CRCs in both locations to better understand the disease. This study hypothesizes that rsCRC and ocCRC differ in P16 and P57 IHC expression and clinicopathological features. It is the first study to compare P16 and P57 expression in rsCRCs to ocCRC.

## Materials and methods

Study design

A retrospective cross-sectional study compared P16Inka4a and P57KIP2 IHC expression in rsCRC versus ocCRC cases using tissue microarray (TMA) slides.

Specimen and data collection

Specimen collection was obtained from paraffin-embedded tissue blocks at the Surgical Pathology Laboratory, the Gastroenterology Center, Mansoura University, Egypt, from 2007 to 2013. CRC cases were grouped into the rsCRC group and the ocCRC group. The rsCRC location in the study describes CRC that occurs in the sigmoid colon, the rectum, or the rectosigmoid junction.

All available clinical, pathological, and follow-up outcomes after diagnosis to the end of the data collection period, including disease-free survival (DFS) and overall survival (OS) in months and recurrence and mortality status for the cases, were received de-identified from patients' personal information.

Two pathologists independently confirmed the diagnosis and the pathological findings of the collected CRC cases on hematoxylin and eosin (H&E) slides. The exclusion criteria for specimens were cases entirely composed of mucinous pools with very few epithelial cells to evaluate on microscopic examination and cases that received preoperative cancer therapy.

TMA construction

Three manual TMA blocks were constructed using the mechanical pencil tip technique [19]. Three representative cores were punched from each case; each was 0.8mm in diameter. Four µm thickness sections from the TMA blocks were used for routine H&E and IHC staining.

IHC staining

Tissue sections on charged slides for immunohistochemistry were auto-deparaffinized and stained using a Leica Bond-III auto-stainer (Leica Biosystems, Newcastle-Upon-Tyne, UK). IHC staining was carried out with anti-P16Ink4a (CINtec p16 Histology mouse monoclonal antibody, Catalog No. 805-4713, Roche, Rotkreuz, Switzerland, undiluted) and anti-P57(KIP2) (Novocastra mouse monoclonal Antibody for human p57 protein, product code: NCL-p57, Leica Biosystems Newcastle Ltd, Newcastle, UK, dilution 1:20) primary antibodies. The manufacturer's instructions for staining were followed. Expression was detected using a streptavidin-biotin method through Leica Bond Refine Detection Kit Catalog No. DS9800. All slide-processing procedures included both positive and negative controls. Positive controls were tonsillar and placental tissues for P16 and P57, respectively. The omission of the primary antibody was used as a negative control for nonspecific staining with a secondary antibody [20]. Mayer's hematoxylin was used to counterstain the nuclei.

IHC evaluation

The IHC-stained sections were examined using an Olympus microscope (BX-53; Olympus Corporation, Tokyo, Japan). The immunostaining was scored blindly for each marker in three high-power fields (HPF) by two pathologists. The average percentage of immunolabel-positive cells was determined in highly stained (i.e., hot-spot) areas. Positive staining for P16 was interpreted as the presence of a brown nucleo/cytoplasmic stain of cells [15]. Semi-quantitative scoring was performed for the percentage of stained cells on a scale from 0 to 3 (0=absent; 1=weak; 2=moderate; 3=strong). The P16 expression levels were defined as positive (overexpression) and negative (hypoexpression), with a cutoff point of 50% of moderately or strongly stained cells [16,21]. Brown nuclear or cytoplasmic immunoreactivity was considered as positive staining for P57 [13,22]. P57 expression was classified as ≤5% and >5% positive cells [22]. In case of borderline positivity of the stained specimen or conflicting results, a third pathologist examined the cases and a consensus was reached.

Sample size calculation

The minimum sample size required for the study was calculated using the formula ((1.96)^2^×pq)/d^2^, in which "p" is the prevalence of P16 in CRC and was considered as 80% [23], "q" is 1-p, and "d" is 20% of the prevalence. As a result, at a confidence interval (CI) of 95%, the minimum sample size is about 25 for each group.

Statistical analysis

All collected data of the study were tabulated, verified, and fed for statistical analysis to Statistical Package for Social Science (IBM Corp. Released 2017. IBM SPSS Statistics for Windows, Version 25.0. Armonk, NY: IBM Corp). Quantitative data were described in mean and standard deviation for normally distributed variables. The median and interquartile ranges were used to describe data if the normal distribution was violated. Shapiro Wilk's test was used to evaluate the normal distribution of quantitative variables. The qualitative categorical variables were expressed in frequency and percentage. The comparative analysis between rsCRC and ocCRC cases regarding demographics and clinicopathological features, including tumor markers, was performed using a two-sample t-test or Mann-Witney test for continuous data according to normality, while for categorical data, a Chi-square test, Fisher's exact test, or Monte Carlo exact had been used as appropriate. Cox proportional-hazards regression models were used to investigate the hazards of disease recurrence and mortality associated with the CRC site (rsCRC and ocCRC) and tumor markers (P16 and P57). P-values ≤0.05 were considered statistically significant.

Quality measures

The recommendations of Meyerholz and Beck for scoring molecular biomarkers in tissue stains [20] and the Reporting Recommendations for Tumor Marker Prognostic Studies (REMARK) guidelines [24] were followed in the current study to increase the reproducibility of scoring results and enhance the comparability of results among studies investigating.

## Results

One hundred fifty CRCs were collected and distributed into two groups; the rsCRC had 86 (57.3%) cases, and the ocCRC constituted 64 (42.7%) cases.

On comparing both groups regarding the clinicopathological data collected, the rsCRC group had a statistically significant lower age group <40 years (P=0.002) and smaller size as measured by the greatest tumor dimension (P=0.02) compared to the ocCRC group. It showed a significantly higher perineural invasion on microscopic examination (P=0.008) and a higher percentage of mismatch repair (MMR) proficient status (P=0.003) than the other site group (Table 1).

**Table 1:** A comparative analysis of the clinicopathological data collected for the rectosigmoid versus other colonic locations colorectal carcinomas (CRC) cases of the study (n=150). CRC: colorectal carcinoma; t: Independent t-test; χ2: Chi-square test; FE: Fischer's exact test; MC: Monte Carlo exact test; U: Mann-Whitney U test; IQR: interquartile range; SD: standard deviation; Min: minimum; Max: maximum; Cm: centimeter; FAP: familial adenomatous polyposis; NOS: not otherwise specified; P: value of statistical significance; *P: ≤0.05 (statistically significant)

Clinicopathological feature		Rectosigmoid CRC (n=86 cases)		Other-colonic location CRC (n=64 cases)		Test of significance (test value)	P-value
		No.	%	No.	%		
Age (Years)	Mean ± SD.	51.2±14.50		54.7±10.71		t (1.705)	0.090
	Min.-Max.	20.0-80.0		25.0-78.0			
Age group (Years)	<40 years	26	30.2	6	9.4	χ^2 ^(9.512)	0.002*
	>40 years	60	69.8	58	90.6		
Sex	Male	51	59.3	42	65.6	χ^2 ^(0.623)	0.430
	Female	35	40.7	22	34.4		
Histological tumor type	Adenocarcinoma, NOS	45	52.3	30	46.9	χ^2 ^(4.198)	0.123
	Mucoid adenocarcinoma	27	31.4	29	45.3		
	Signet ring carcinoma	14	16.3	5	7.8		
Histological tumor grade	Grade I	7	8.1	9	14.1	χ^2 ^(3.389)	0.184
	Grade II	31	36.0	15	23.4		
	Grade III	48	55.8	40	62.5		
Tumor size by greatest dimension (cm)	Mean ± SD.	6.1±2.87		7.2±2.95		t (2.357)	0.020*
	Min.-Max.	2.0-15.0		2.5-17.0			
Gross tumor morphology	Fungating	29	33.7	32	50.0	χ^2 ^(5.216)	0.074
	Ulcerating	32	37.2	14	21.9		
	Annular	25	29.1	18	28.1		
Multiplicity of the tumor	Negative	78	90.7	56	87.5	χ^2 ^(0.394)	0.530
	Positive	8	9.3	8	12.5		
Histological pattern of tumor edge	Advancing (budding)	79	91.9	58	90.6	χ^2 ^(0.071)	0.790
	Pushing	7	8.1	6	9.4		
Lymphovascular emboli status in the tumor	Negative	25	29.1	28	43.8	χ^2 ^(3.461)	0.063
	Positive	61	70.9	36	56.3		
Perineural invasion status in the tumor	Negative	51	59.3	51	79.7	χ^2 ^(7.007)	0.008*
	Positive	35	40.7	13	20.3		
Peritumoral lymphocytic response status (Crohn-like response)	Negative	63	73.3	46	71.9	χ^2 ^(0.035)	0.851
	Positive	23	26.7	18	28.1		
Intratumoral lymphocytic response status (tumor-infiltrating lymphocytes)	Negative	83	96.5	60	93.8	FE	0.460
	Positive	3	3.5	4	6.3		
Excessive peri and intratumoral neutrophilic infiltrate status	Negative	43	50.0	30	46.9	χ^2 ^(0.143)	0.705
	Positive	43	50.0	34	53.1		
Associated adenoma	Not on top of adenoma	47	54.7	33	51.6	χ^2 ^(0.141)	0.708
	On top of adenoma	39	45.3	31	48.4		
Associated Bilharziasis	Negative	76	88.4	51	79.7	χ^2 ^(2.132)	0.144
	Positive	10	11.6	13	20.3		
Associated FAP	Negative	83	96.5	60	93.8	FE	0.460
	Positive	3	3.5	4	6.3		
Depth of tumor invasion (T)	T1	2	2.3	1	1.6	MC	0.344
	T2	13	15.1	8	12.5		
	T3	62	72.1	53	82.8		
	T4	9	10.5	2	3.1		
Lymph Node staging (N)	N0	33	38.4	32	50.0	χ^2 ^(4.293)	0.117
	N1	24	27.9	20	31.3		
	N2	29	33.7	12	18.8		
Distant metastasis status (M)	M0	82	95.3	63	98.4	FE	0.394
	M1	4	4.7	1	1.6		
TNM stage	Stage I	9	10.5	9	14.1	MC	0.399
	Stage II	23	26.7	23	35.9		
	Stage III	50	58.1	31	48.4		
	Stage IV	4	4.7	1	1.6		
Surgical cut margins status	Free	79	91.9	63	98.4	FE	0.139
	Infiltrated	7	8.1	1	1.6		
Mismatch repair proteins (MMR) status	MMR proficient	64	75.3	32	50.8	MC	0.003*
	Loss of MLH1 and MLH6 proteins	1	1.2	4	6.3		
	Loss of MSH2 and MSH6 proteins	5	5.9	13	20.6		
	Other protein loss	15	17.6	14	22.2		
	Total MMR data retrieved	85	100.0	63	100.0		
Recurrence status on follow-up	Free	65	75.6	50	78.1	χ^2 ^(0.133)	0.716
	Recurrence	21	24.4	14	21.9		
Disease-free (DFS) survival (months)	Mean ± SD.	34.4±23.13		41.6±23.34		U (2038.0)	0.030*
	Min.-Max.	6.0-77.0		5.0-79.0			
	Median (IQR)	25.5 (14.0-62.0)		36.0 (18.0-64.0)			
Mortality outcome on follow-up	Alive	72	83.7	47	73.4	χ^2 ^(2.367)	0.124
	Death	14	16.3	17	26.6		
Overall survival (months)	Mean ± SD.	36.2±21.47		44.1±21.52		U (2111.5)	0.015*
	Min.-Max.	5.0-77.0		10.0-79.0			
	Median (IQR)	28.5 (18.0-61.25)		43.0 (23.0-64.0)			

There was no statistically significant difference between the two groups regarding sex, histological type, grade, stage, gross shape, multiplicity, tumor edge shape, lymphovascular invasion, tumoral inflammatory response status, or the presence/absence of an associated lesion such as familial adenomatous polyposis coli, adenoma, or bilharziasis (Table 1).

Using the Cox regression model, there was a statistically nonsignificant increased hazard of disease recurrence among rsCRC patients by about 1.3 folds compared to the ocCRC group of patients (HR=1.274, 95% CI: (0.523-3.105), p=0.594). Furthermore, there was a statistically nonsignificant increased hazard of mortality among rectosigmoid patients by about 1.1 folds compared to the other sites group of patients (HR=1.127, 95% CI: (0.478-2.66), p=0.784).

On IHC preparation, tissues of five rsCRC and five ocCRC cases stained for P16Ink4a were lost (n=81 and n=59, respectively; total=140 cases). Moreover, tissues of five rsCRC and six ocCRC cases stained for P57KIP2 were lost (n=81 and n=58, respectively; total=139 cases).

**Figure 1:**
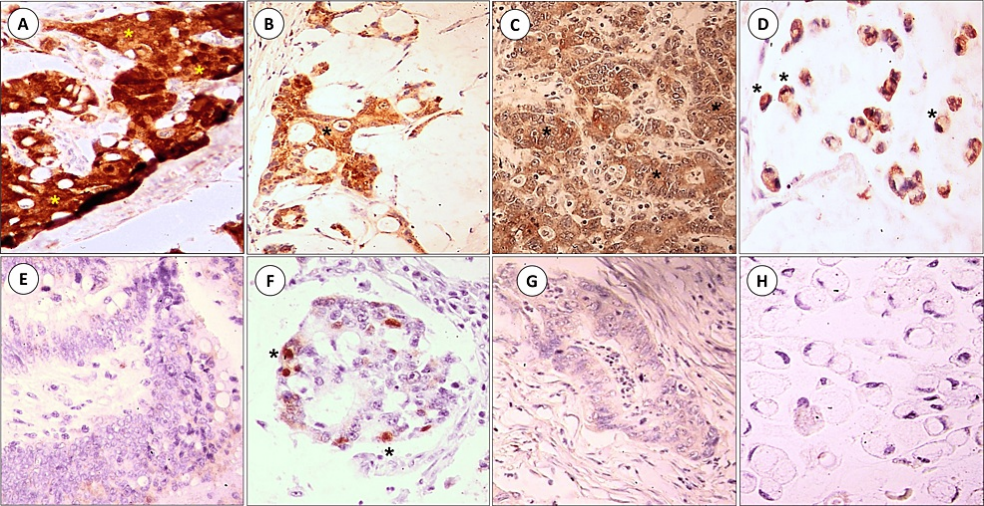
Immunohistochemical expression of P16Ink4a and P57KIP2 in rectosigmoid (A-D) colorectal carcinoma (CRC) and CRC of other-colonic locations (E-H) (original magnification X400). Immunohistochemical expression (starred) of P16Ink4a and P57KIP2 in rectosigmoid CRCs (A: P16Ink4a, adenocarcinoma, NOS; B: P16Ink4a, mucoid adenocarcinoma; C: P57KIP2, adenocarcinoma, NOS; D: P57KIP2, Signet ring carcinoma) is higher than their expression in CRCs of other colonic locations (E: P16Ink4a, adenocarcinoma, NOS; F: P16Ink4a, mucoid adenocarcinoma; G: P57KIP2, adenocarcinoma, NOS; H: P57KIP2, Signet ring carcinoma) (original magnification X400). NOS: not otherwise specified; P: protein, CRC: colorectal carcinoma

The pattern of P16 IHC expression was nucleo/cytoplasmic in all cases, while P57 showed cytoplasmic expression in the majority of cases (96.9%, n=63 out of 65 total positive cases in both study groups). Only two cases showed a combined nucleocytoplasmic P57 expression (3.1%, n=2 out of 65 total positive cases), one in each study group. Using the Chi-square test to compare the IHC expression of P16Ink4a and P57KIP2 proteins in rsCRCs versus ocCRCs stained tissues, the rsCRC group showed significantly higher positive expression of both markers (P=0.02, P=0.03, respectively) (Figure 1 and Table 2).

**Table 2:** A comparison of the immunohistochemical expression status for P16Ink4a and P57KIP2 proteins in rectosigmoid versus other-colonic location colorectal carcinoma (CRC) in the study groups. CRC: colorectal carcinoma; χ^2^: Chi-square test; P: value of statistical significance; * p≤0.05 (statistically significant)

Studied protein expression	The immunohistochemical protein expression status	Rectosigmoid CRC		Other-colonic location CRC		Test of significance (value)	P-value
		No.	%	No.	%		
P16Ink4a immunohistochemical expression	P16Ink4a positive expression	39	48.1	17	28.8	χ^2 ^(5.317)	0.021*
	P16Ink4a negative expression	42	51.9	42	71.2		
	P16Ink4a total examined cases	81	100.0	59	100.0		
P57KIP2 immunohistochemical expression	P57KIP2 positive expression	44	54.3	21	36.2	χ^2 ^(4.455)	0.035*
	P57KIP2 negative expression	37	45.7	37	63.8		
	P57KIP2 total examined cases	81	100.0	58	100.0		

Using Cox regression to assess the tumor markers' relationship to the disease outcome in the rsCRC group, P16 and P57 showed no statistical significance for the hazard of disease recurrence or mortality. For the disease recurrence hazard, P16 showed a statistically nonsignificant increase among positive P16 patients by about 4.0 folds compared to the negative P16 group of patients (HR=4.021, 95% CI: (0.955-16.930), p=0.058), while P57 expression showed a statistically nonsignificant reduction in the hazard for disease recurrence (HR=0.360, 95% CI: (0.091-1.425), p=0.145).

Similarly, the hazard of mortality increased among positive P16 patients by about 2.6 folds compared to the negative P16 group of patients (HR=2.557, 95% CI: (0.586-11.157), p=0.212), contrary to P57, which showed a reduced mortality hazard with expression (HR=0.236, 95% CI: (0.053-1.048), p=0.058).

In the ocCRC group, for the hazard of disease recurrence, P16 expression showed a significantly increased hazard by about 8.2 folds among positive patients compared to the negative P16 group of patients (HR=8.196, 95% CI: (1.766-38.034), p=0.007*). On the contrary, P57 showed a nonsignificant hazard reduction with disease recurrence (HR=0.793, 95% CI: (0.197-3.198), p=0.744).

The hazard of mortality in the ocCRC group increased significantly among positive P16 patients by about 5.6 folds compared to the negative group of patients (HR=5.574, 95% CI: (1.11-28.0), p=0.037*). In contrast, P57 showed a significant reduction in mortality hazard due to the disease (HR=0.121, 95% CI: (0.019-0.790), p=0.027*).

## Discussion

Studies started to call for a CRC classification by specific location rather than tumor-sidedness [3,7]. Though not fully elucidated, the rectosigmoid location for cancers has been recently described as having unique clinicopathological criteria and therapeutic challenges [7,8]. As calls that recommend widening the application of precision medicine and personalized CRC treatments with better therapeutic outcomes are recently increasing [2], unraveling protein expression differences in rsCRC compared to ocCRC locations helps understand tumor pathogenesis and prognosis and design personalized therapeutic and follow-up plans.

The literature reported differences in CRC criteria according to tumor sidedness. Few reports investigated the criteria of rectosigmoid tumors compared to other colonic locations. The current study investigated P16 and P57 IHC expression in rsCRC versus ocCRC and their relationship to the clinical outcome in both groups. It hypothesized that rsCRC and ocCRC differ in P16 and P57 IHC expression and clinicopathological features. Our study showed that the rsCRC cases had a significantly lower age <40 years, higher microsatellite stability, and frequency of perineural invasion, with lower DFS and OS. Significantly higher positive P16Ink4a and P57 IHC expression was found in the rsCRCs compared to the ocCRCs; however, their relationship to recurrence and mortality hazards in the rsCRCs was statistically nonsignificant. In the ocCRCs, P16Ink4a positivity was significantly associated with a higher disease recurrence and mortality hazard, while P57KIP2 positivity was significantly associated with a lower mortality hazard.

Like our study, other studies described an increasing incidence of rsCRCs in patients younger than 40, though the exact explanation for this association is still unclear [25].

In our study, the OS and DFS were lower in the rsCRC group, with higher mortality and recurrence hazards. Some studies reported the presence of the CRC tumor on the left side to be an independent bad prognostic factor for the disease outcome [26]. Moreover, a possible explanation for the lower survival in the rsCRC group in our study was the higher frequency of MMR-proficient (i.e., microsatellite stable (MSS)) status in the rsCRC. Other studies reported better survival with the higher frequency of MMR-deficient CRCs (i.e., microsatellite instability (MSI)), with right-sided CRCs showing a higher MSI frequency than left-sided cancers [27]. In addition, in our study, perineural invasion frequency was higher in the rsCRC group, which might explain the lower survival and higher mortality reported. Many studies reported a significant association between perineural invasion and bad CRC prognosis and outcome, including higher recurrence, lower DFS, and OS, requiring adjuvant therapy [28]. Though no reports in the literature described specifically the perineural invasion status in the rsCRCs versus ocCRCs, perineural invasion reports are higher in rectal cancer compared to colon cancer and reflect a bad prognosis [28].

In the current study, P16 positivity was found to be a bad predictor of survival and recurrence, while P57 positivity is a good prognostic survival factor in the ocCRC group. The different P16 and P57 expression patterns and prognostic values in rsCRC compared to ocCRC reflect different pathogenesis of both CRC groups.

P16 functions as a tumor suppressor, acting through CDK4 and CDK6 to keep the retinoblastoma (Rb) gene product hypophosphorylated, hence inactivated, pausing the cell cycle [11,18]. P16-Rb signaling is one of the most frequently altered cancer pathways [11]. P16 IHC expression is absent in most normal human tissues, including the colonic tissue [29], which makes it a good target for investigation as a predictive factor and therapeutic modulation. Studies investigating the differential P16 protein expression in different CRC anatomic locations are deficient. Most reports describe P16 expression in CRC irrespective of site consideration or tumor-sidedness [16,29]. In the current study, P16 positivity was higher in rsCRC than in ocCRC types. Similar results regarding the expression of P16 in distal CRC sites were noticed in previous studies [16,21,23]. As P16Ink4a can be used to investigate high-risk HPV infection in various tissues, some studies related the high P16Ink4a expression in CRCs to high-risk HPV infection of these tumors [16,21]; however, P16Ink4a expression in tumors is not restricted to HPV positivity and confirmation by detection of HPV DNA is needed for such assumption [21].

De Wispelaere et al. reported a significant association between P16 positivity and the MMR status in CRCs with a higher percentage of P16-positive expression in the MMR-proficient group compared to the MMR-deficient (i.e., MSI) groups [29]. MSI status leads to an upregulated methylation of the P16 gene, resulting in a reduction of P16 expression [29], which explains the lower P16 expression in the ocCRC group.

The prognostic value of P16 expression status in CRCs is conflicting. It has not yet been confirmed in the literature [29]. Like our study, some reported a poorer CRC prognosis with P16 overexpression [29]. In contrast, other studies described the reduced expression as being associated with poor prognosis [30], while others reported no significant relationship between survival and P16 expression [21].

An explanation of how P16 expression is associated with poor cancer prognosis despite its physiological role as a tumor suppressor in the cell cycle was provided in the literature. Researchers described two mechanisms: the expression of viral oncoproteins in cases of HPV infection and the loss of Rb protein in cancer leading to oncogenic stress with compensatory P16 activation, which fails to arrest tumor progression due to the dysfunctional downstream Rb in the P16-Rb pathway [9]. These mechanisms are more likely to occur in cancers than P16 mutations and methylation [9,21]. Cytoplasmic expression of P16 reflects an active P16 protein status [21]. Our study demonstrated nucleo/cytoplasmic staining in all cases.

The association of P57 with different CRC locations or sidedness has not yet been investigated in the literature. Our study showed a higher P57 in the rsCRC group. Yet, P57 positivity was associated with significantly lower mortality and a statistically nonsignificant lower disease recurrence in the ocCRC group rather than the rsCRC group. P57 controls the cell cycle transition from G1 to S phases. Moreover, it regulates cytoskeletal dynamics, apoptosis, and cellular senescence [9,11]. In our study, the majority of cases demonstrated cytoplasmic P57 expression. Cytoplasmic P57 expression was reported to have similar roles as nuclear P57 expression in cancers, including reducing the motility of cancerous cells, stabilizing the actin cytoskeleton, inhibiting apoptosis, and suppressing invasion and metastasis [9,13].

This study is the first comparative analysis of the IHC expression of P16 and P57 in rsCRC compared to ocCRC and their relatedness to the outcome in each group. Based on our results and literature findings [3], we propose that the rectosigmoid location of cancers constitutes a group with criteria different from other colonic locations, and investigating it as a separate group rather than investigating CRC based on tumor-sidedness will yield more positive findings. Study limitations included a relatively limited number of cases recruited from a single lab. Further multicenter research that consists of a higher number of cases is recommended. Moreover, further research comparing P16 and P57 expression in rsCRC, ocCRC, and normal colonic tissue from corresponding locations will add valuable insights into understanding these proteins' role and their modulation effect in personalized CRC therapy.

## Conclusions

rsCRC differs from CRC in other colonic locations in clinicopathological criteria and protein expression patterns. Both P16Ink4a and P57KIP2 IHC expressions are higher in the rsCRC compared to CRC in other colonic locations. However, the value of these markers as outcome predictors is higher in the CRC of colonic sites other than the rectosigmoid locations. P16Ink4a positivity is linked to worse outcomes in terms of OS and DFS. In contrast, the P57 positivity denotes better OS in CRC of locations other than the rectosigmoid. So, P16Ink4a and P57KIP2 modulation may be therapeutic in these locations. Research on the differential modulation of P16Ink4a and P57KIP2 expression according to specific colonic location is needed, which may improve CRC clinical outcomes.
